# Motives for leisure-time physical activity participation: an analysis of their prevalence, consistency and associations with activity type and social background

**DOI:** 10.1186/s12889-023-17304-0

**Published:** 2023-12-02

**Authors:** Karsten Elmose-Østerlund, Birgitte Westerskov Dalgas, Thomas Viskum Gjelstrup Bredahl, Lars Lenze, Jens Høyer-Kruse, Bjarne Ibsen

**Affiliations:** 1 Centre for Sports, Health and Civil Society, Research Unit for Active Living, Department of Sports Science and Clinical Biomechanics, University of Southern Denmark, Odense, Denmark; 2 Research Unit for Active Living, Department of Sports Science and Clinical Biomechanics, University of Southern Denmark, Odense, Denmark; 3 Institute of Sport Science, University of Bern, Bern, Switzerland; 4 School of Education, University of Applied Sciences and Arts Northwestern Switzerland, Windisch, Switzerland

**Keywords:** Sports participation, Exercise, Leisure, PALMS, Motivation, Psychology, Gender, Age, Educational level

## Abstract

**Background:**

Studies argue that knowledge about motives for physical activity participation can inform activities, initiatives and interventions to promote physical activity. However, most of these studies are based on small sample sizes and only include participants within a few selected types of PA. Further, they have not examined the consistency of individuals’ motives across different activity types. As a result, this article examines RQ1: the most prevalent motives, RQ2: the association of motives with activity type and social background characteristics, and RQ3: the consistency of motives across different activity types.

**Methods:**

We utilised data from a survey of physical activity participation among 163,000 adult Danes (aged 15 + years). In the survey, the participants were asked about their participation in thirteen activity types and about their motives for practising the activity types they reported to do at least weekly. The motive items were operationalised based on the eight dimensions in the Physical Activity and Leisure Motivation Scale (PALMS). We conducted analyses of mean values and standard deviations as well as multilevel regression analyses.

**Results:**

We identified large differences in the importance of different motives for physical activity participation. The three most important motives were psychological condition (M = 4.54), physical condition (M = 4.48) and enjoyment (M = 4.36). We also found significant associations between motives and activity types in particular, but also between motives and social background characteristics (gender, age and educational level). For instance, we found that compared to running, physical condition was a much less important motive in outdoor activities (b = -3.01), activities on water (b = -2.44) and street sports (b = -2.38). Finally, our analysis showed how individuals’ motives are not consistent across different activity types.

**Conclusions:**

Our study contributes to the literature on motives for physical activity participation by using a large sample of individuals and by differentiating motives according to a wide range of activity types. The results underline the need to study motives in relation to activity types, as there are large differences in the prevalence of different motives. Our findings suggest that motives are not consistent across activity types, but rather they develop in an interplay between the individual and the activity type practised.

**Supplementary Information:**

The online version contains supplementary material available at 10.1186/s12889-023-17304-0.

## Background

With an increasingly sedentary lifestyle followed by rising obesity levels and increases in lifestyle diseases [1], the need to get more people to be (more) physically active is evident. Regular physical activity (PA) participation has been found to have the potential to promote good physical, mental and social health [2–5] and to contribute to general well-being [5, 6].

With the multifaceted benefits of PA being well-established in the current literature, it seems relevant to increase our understanding of why some people are physically active, while others are not. Reviews of the existing literature have shown how this is a complex undertaking, and that several factors at different levels, including the intrapersonal, individual and environmental levels, have been identified as correlates or determinants of PA participation [7]. However, the reviews underline the centrality of psychological factors regarding PA participation [8–10].

A relevant psychological factor is people’s rationales for PA participation, typically addressed as motives [11, 12]. Studies argue that knowledge about the relative importance and diversity of different motives for PA participation can inform activities, initiatives and interventions to promote PA participation in the population generally, within selected subgroups and in connection to different activity types [10, 13–17].

A validated tool for studying motives for PA participation is the Physical Activity and Leisure Motivation Scale (PALMS). PALMS was developed to bridge the gap between the application of different theoretical frameworks and atheoretical approaches to the study of motives for PA participation [15]. The PALMS framework emanated from a qualitative interview study [18], where the identified themes aligned with constructs of self-determination theory, namely intrinsic-extrinsic motivation, and also with items and factors identified in previous studies. Studies have found PALMS to be a reliable and valid instrument for measuring motives for any kind of PA [15, 19, 20] and across different cultural contexts [20–23].

The PALMS framework describes eight dimensions of motives for PA participation, including mastery, physical condition, affiliation, psychological condition, appearance, others’ expectations, enjoyment and competition/ego. Studies have found physical condition, enjoyment and mastery to be the most prevalent motives for PA participation, while others’ expectations have consistently been ranked as the least prevalent motive [19, 24, 25]. Some studies have also found psychological condition to be a prevalent motive for PA participation [24, 25].

Some of the studies that utilise the PALMS framework also examine differences in the relative importance of the motives according to social background (gender and age) and activity type. In relation to gender, competition/ego was generally found to be a more important motive for men than women, while appearance was rated higher among women than men [19, 24, 25]. A couple of studies also found that men on average rated affiliation as a more important motive for their PA participation than women [19, 24].

Regarding age, the identified studies apply dissimilar age differentiations. One study identified negative associations between increasing age on the one hand and the motives appearance and competition/ego on the other [25]. Another study differentiated the motives of young adults (20–40 years) from the motives of middle-aged adults (21–64 years) and found affiliation, mastery, enjoyment and competition/ego to be more important for young adults. Conversely, psychological condition and others’ expectations were rated higher among middle-aged adults compared to young adults [24].

Besides social background, some studies have examined differences in motives between activity types. Generally, these studies find that motives variate significantly according to the activity type practised [15]. Even though it is difficult to compare findings across studies due to different operationalisations of activity type, there is some consistency in the findings, for instance when differentiating between exercise activities (e.g. fitness and running) and team sports activities (e.g. football, hockey and Australian football). While psychological condition is rated higher among participants in exercise activities, mastery and competition/ego are more important motives for team sports participants [24–26]. There is also some evidence that appearance is a more important motive in exercise activities [24, 26], and that physical condition is more important for exercise sports participants, while affiliation is more important for team sports participants [24, 25].

One study includes racket sports in the analysis and finds that mastery and competition/ego are relatively important for the participants in this activity type [24], while another study that includes tennis finds enjoyment to be a relatively important motive for tennis players [26]. Finally, a study that includes yoga in the analysis finds yoga participants to be more motivated by psychological condition and less by affiliation, competition/ego and physical condition [26].

Despite the multifaceted and interesting findings presented above, most of the studies that have examined motives for PA participation share common limitations. First, they rely on relatively small sample sizes, and, second, they lack systematically sampled participants to cover the breadth of age groups and activity types that would allow them to test predictions about social background and activity type differences [15]. Further, it is a recommendation across many of the studies on motives for PA that the knowledge they generate can be used to recommend specific activity types to individuals based on their composition of motives [15, 19, 25]. However, this builds on the assumption that individuals’ motives are consistent across activity types, and we were not able to identify any studies that had tested this assumption empirically.

To address the knowledge gaps in studies on motives for PA participation, this article will make use of data from a large-scale survey to enable statistically valid analyses on the similarities and differences in motives for a broad range of activity types and according to different social background characteristics. More specifically, this article will answer the following three research questions: RQ1: Which motives are particularly prevalent for PA participation? RQ2: How are activity type and social background (gender, age and education) associated with motives for PA participation? RQ3: To what extent are individuals’ motives for PA participation consistent across different activity types? Our analyses will inform and nuance our understanding of motives for PA participation, which has implications for research on motives and might aid sport and health professionals in designing and tailoring suitable and effective activities, initiatives and programmes that seek to increase PA participation.

## Methods

This article utilises data from the ‘Moving Denmark’ project that examines PA participation among adult Danes.

### Data collection

We draw on survey data collected in October and November 2020. The survey was distributed using digital mail (‘e-Boks’), which is a tool for safe communication between citizens and public authorities in Denmark. The survey population consisted of all adults aged 15+ years living in Denmark who possessed a Danish social security number. Before sampling, the survey population was stratified according to the size of their municipality. To collect reliable statistical data regarding all 98 Danish municipalities, proportionally higher numbers of individuals were sampled from the smallest municipalities, while proportionally lower numbers were sampled from the biggest municipalities. A total of 404,452 individuals were sampled, of whom 163,133 responded to the survey, equating to a response rate of 40 per cent. The response rate was a bit higher among women compared to men, and among the elderly compared to young people. As a result, the data were weighted according to gender and age.

The survey focused on PA participation in four domains: home, work, transportation and leisure time. Respondents were asked about their motives for PA participation, evaluated local opportunities for being active (e.g. sports facilities, green areas), reported their health status, and provided information about their social background. To increase the reliability of the survey, we opted to ask the respondents about their participation in specific activity types rather than asking generally about their PA participation. This leaves less room for interpretation, which improves the consistency of our measurements. The validity of the survey was ensured through validation, including expert validation, the use of cognitive interviews and piloting before distribution. After data collection, the dataset was supplemented with information from Statistics Denmark regarding the respondents’ social background.

### Datasets

Of the 163,133 respondents, 140,045 participated in at least one activity type at least once a week in their leisure-time, and their responses constitute our original dataset. However, because the questions regarding motives were asked in the context of different activity types, we transformed the dataset from a wide to a long format to enable analyses that include activity type and social background in the same statistical model. While each respondent appears as one observation in the original wide dataset, the respondents appear as observations according to the number of activity types they practise at least weekly in the transformed long dataset. Because of this, the long dataset contains 385,631 observations, representing the total number of activity types practised at least once a week by the 140,045 respondents. Table 1 presents the number of respondents who practise the thirteen surveyed activity types at least once a week.

**Table 1:** Thirteen activity types included in survey and number of respondents

Activity type	*N*
Running	32,654
Walking and hiking	115,870
Biking	56,891
Fitness (e.g. weight training, cardio workout, team workout)	62,165
Mental/flexibility/stability training (e.g. yoga, Pilates, meditation/mindfulness)	29,166
Team ballgames (e.g. football, handball, volleyball, beach volley, floorball)	9,906
Other ballgames (e.g. golf, badminton, table tennis, tennis, squash)	13,776
Gymnastics (e.g. jump gymnastics, rhythmic gymnastics, apparatus work)	14,116
Dance (e.g. partner dance, fitness dance, modern dance, creative dance, street dance)	9,503
Activities in water (e.g. swimming, diving, pool training, winter swimming)	19,388
Activities on water (e.g. canoeing/kayaking, rowing, sailing, surfing, stand up paddle)	4,564
Outdoor activities (e.g. outdoor life, fishing, hunting, scouts, role-playing games)	15,594
Street sports (e.g. roller skating, scooters, skateboarding, parkour, street basketball)	2,038
Total	385,631

Note: The survey included four additional activity type categories: ‘rehabilitation’, ‘physically active games and play’, ‘other sporting activities’ and ‘other physically active leisure activities’. They are not included in the table, because the motive questions were not asked regarding these activity types

Table 2 compares the distribution of observations according to gender, age and educational level between the wide and long dataset. The differences between the two datasets are relatively modest. However, the percentages of women, people with tertiary education and people aged 15–39 years are slightly higher in the long dataset compared to the wide dataset.

**Table 2:** Distribution according to gender, age and educational level in the two datasets

	**Long dataset (*N* = 385,631)**		**Wide dataset (*N* = 140,045)**	
**Variable**	**%**	**N**	**%**	**N**
Gender: man	42.9	165,547	44.3	61,992
Gender: woman	57.1	220,084	55.7	78,053
Age: 15–24	12.4	47,926	10.6	14,816
Age: 25–39	16.9	65,313	16.8	23,547
Age: 40–59	34.6	133,371	35.0	48,991
Age: 60 +	36.1	139,021	37.6	52,691
Education: primary	17.7	68,421	18.1	25,417
Education: secondary	39.6	152,876	41.3	57,796
Education: tertiary	42.0	161,787	39.9	55,813

### Measurement instruments, variables and transformations

In the preparatory phase, articles that drew on the PALMS framework were screened, and different operationalisations of the eight dimensions in the PALMS framework served as inspiration for the operationalisations applied in the survey, which are shown in Table 3. The respondents were asked for their (dis)agreement with the motive items from 1 = ‘completely disagree’ to 5 = ‘completely agree’.

**Table 3:** Operationalisation of eight motive dimensions in PALMS framework in survey

PALMS motive dimension	Operationalisation ‘I perform the activity…’
Mastery	To get better at the activity
Physical condition	To maintain or improve my health
Affiliation	To spend time with others
Psychological condition	To do something nice for myself
Appearance	To maintain or improve my appearance
Others’ expectations	Because others in my social circle encourage me to do so
Enjoyment	Because I like the activity
Competition/ego	To compete with myself or others

To examine activity type participation, the respondents were first asked to indicate whether they had practised any of the activity types shown in Table 1 within the past year. Next, they were asked to indicate the specific activity or activities that they had practised within each activity type (e.g. whether they had practised football, handball, volleyball etc. within the activity type ‘team ballgames’). Lastly, they were asked to indicate how often they had practised the selected activities. Respondents who participated in at least one activity at least once a week were presented with the motive items displayed in Table 3 regarding the activity type in question. The activity type variables were utilised for the statistical analyses as binary variables, with the value 1 indicating that the activity type was practised at least once a week, while 0 indicated that this was not the case.

As indicators of social background, we included gender, age and educational level. Gender and age information was extracted from social security numbers. Age was recoded from a continuous variable to an ordinal variable with four categories: 15–24, 25–39, 40–59 and 60 + years to represent different life stages such as adolescence and young, middle and late adulthood. Educational level was coded based on information from Statistics Denmark, which allowed respondents to be grouped into three categories, matching the categories of primary, secondary and tertiary education.

### Data analyses

We conducted the statistical analyses for this article in three steps representing our three research questions. To address RQ1, we examined the prevalence of the different motives by calculating mean values and standard deviations.

To examine RQ2, associations between activity type and social background on the one hand and motives for PA on the other were examined through statistical multilevel regression analyses. We decided to follow a multilevel approach to be able to consider the multiple observations (meaning multiple activity types) per individual that are not independent [27]. The eight motive items were included as dependent variables, and since they were measured on a Likert scale ranging from 1 to 5, we opted for a Generalised Linear Mixed Model using the standard formula for multilevel modeling with ordinal outcome variables [28]. Our model was a random intercept model, which enabled the analysis to account for varying intercepts between the individuals included in the analysis. The covariance structure was unstructured, thereby not imposing any constraints on the values calculated. The activity types constituted the independent variables at the first level, and the social background characteristics (gender, age and educational level) constituted the independent variables at the second level. Because the age and education variables were in some instances not monotonously related to the dependent variables, we categorised them in order not to violate the assumptions of ordinal regression analysis. Finally, we checked the covariance between the independent variables and calculated VIF- and Tolerance-values to examine multicollinearity, but we did not identify values outside the acceptable range.

Though we are treating motives as dependent variables and activity type and social background as independent variables, this should not be interpreted as a claim that a causal relationship exists between the choice of activity type and the motives for PA participation. Rather, we are interested in understanding how motives variate according to activity type when controlling for social background and vice versa. In the discussion, we address the causality issue further.

For activity type, running was chosen as the reference category. For age, the youngest age group was chosen as the reference category, and for education, primary education was selected as the reference category.

Because of the high number of observations both within the dataset as a whole and within the different subgroups examined, nearly all results were statistically significant. Therefore, the presentation of the results does not cover all statistically significant results, but rather focuses on selected key results and tendencies in the material based on the magnitude of the non-standardised regression coefficients.

To answer RQ3, we examined the consistency of individuals’ motives for PA participation by calculating differences in the ratings of motives by activity type among the 108,621 respondents who participated in two activity types or more at least once a week. For each respondent, we calculated an average rating for each motive across the two or more activity types practised at least once a week. We then calculated the mean difference by subtracting the rating of each motive connected to the activity types practised from the average rating across all activity types practised by the respondent at least once a week. This allowed us to calculate mean values and standard deviations for the mean differences within individuals across activity types.

To serve as a reference point against which to compare the mean differences in motive ratings within individuals across activity types, we also calculated mean differences in motive ratings across individuals within activity types. For each of the thirteen activity types, we calculated the difference between each respondent’s rating of the motive items and the mean rating of the motive items across all the respondents practising the activity type at least once a week. On this basis, we calculated mean values and standard deviations for the mean differences within each of the thirteen activity types across individuals.

## Results

In this section, we first describe the importance of different motives for PA participation (RQ1). We then present associations between activity types, social background characteristics and different motives for PA participation (RQ2). Finally, we describe the results of our analysis of the consistency of individuals’ motives for PA participation across different activity types (RQ3).

### Prevalence of different motives for PA participation

Three of the eight motives examined stand out in Table 4 as the most prevalent when calculating mean values across all responses, namely psychological condition (M = 4.54), physical condition (M = 4.48) and enjoyment (M = 4.36). By contrast, two motives stand out as the least prevalent, namely others’ expectations (M = 2.59) and competition/ego (M = 2.75). It is worth noting that the three most prevalent motives also show the lowest standard deviations (SD = 0.93 or lower), indicating their general prevalence. By contrast, the standard deviations for the other five motives are somewhat higher (SD = 1.27 or higher), indicating larger differences in the prevalence of these motives. How the mean values and standard deviations variate according to activity type and social background can be viewed in Supplementary Table 1.

**Table 4:** Mean values and standard deviations for eight motive items (*N* = 385,631)

Motive	Mean	Std.dev
Mastery	3.61	1.27
Physical condition	4.48	0.88
Affiliation	3.23	1.42
Psychological condition	4.54	0.80
Appearance	3.37	1.37
Others’ expectations	2.59	1.34
Enjoyment	4.36	0.93
Competition/ego	2.75	1.44

### Associations between motives for PA participation, activity type and social background

In Table 5, all results from the multilevel regression analyses are reported. The intraclass correlation coefficients (ICCs) in the empty models range from 0.33 to 0.61, which justifies the use of multilevel modeling, due to the significant variance found at the second (individual) level across all motive items [29]. As a result, differences in the rating of the motive items are explained by both within- and between-individual differences.

**Table 5:** Results from the multilevel regression analyses conducted with eight motive items as dependent variables (*N* = 385,631)

	**Mastery**	**Physical condition**	**Affiliation**	**Psychological condition**	**Appearance**	**Others' expectations**	**Enjoyment**	**Competition/ego**
**Activity type**								
Running (ref.)								
Walking and hiking	-1.72***	-1.51***	1.30***	-0.87***	-1.10***	0.13***	0.62***	-1.42***
Biking	-1.71***	-1.65***	0.54***	-1.16***	-1.14***	0.03*	0.40***	-1.05***
Fitness	-0.30***	-0.04*	0.65***	< 0.01	0.29***	0.31***	-0.09***	-0.46***
Mental/flexibility/stability training	-0.01	-0.73***	0.20***	0.13***	-1.33***	-0.04*	0.56***	-1.55***
Team ballgames	-0.30***	-1.35***	3.55***	-0.73***	-1.30***	1.53***	1.75***	0.89***
Other ballgames	-0.02	-1.76***	3.45***	-0.85***	-1.59***	1.35***	1.73***	1.39***
Gymnastics	-0.81***	-1.09***	1.88***	-0.66***	-0.69***	0.63***	0.38***	-0.92***
Dance	-0.36***	-1.95***	2.39***	-0.87***	-1.15***	0.78***	1.41***	-1.00***
Activities in water	-1.18***	-1.47***	1.48***	-0.60***	-1.08***	0.60***	0.98***	-0.96***
Activities on water	-0.13***	-2.44***	2.60***	-0.76***	-1.87***	1.07***	1.76***	-0.31***
Outdoor activities	-1.41***	-3.01***	2.16***	-1.24***	-2.31***	0.88***	1.13***	-1.18***
Street sports	-0.58***	-2.38***	1.79***	-1.63***	-1.76***	0.88***	0.95***	-0.57***
**Social background**								
Gender: woman	0.19***	0.60***	0.13***	0.89***	0.86***	0.01	0.51***	-0.46***
Age: 15–24 (ref.)								
Age: 25–39	0.04*	0.40***	-0.06**	0.57***	0.29***	-0.34***	0.33***	-0.09***
Age: 40–59	-0.15***	0.80***	-0.28***	1.00***	-0.08**	-1.05***	0.62***	-0.43***
Age: 60 +	0.21***	1.26***	-0.32***	1.13***	-0.76***	-1.20***	0.65***	-0.35***
Education: primary (ref.)								
Education: secondary	-0.12***	0.20***	-0.07***	0.20***	0.08***	-0.18***	0.14***	-0.14***
Education: tertiary	-0.50***	0.22***	-0.12***	0.15***	-0.09***	-0.33***	0.18***	-0.55***
**Model characteristics**								
Intercept variance (empty model)	1,63***	1,67***	1,74***	2,27***	5,07***	4,09***	1,69***	3,24***
Intercept variance (full model)	1.93***	1.71***	2.04***	2.15***	5.28***	4.07***	1.78***	3.37***
ICC (empty model)	0.33	0.34	0.35	0.41	0.61	0.55	0.34	0.50
ICC (full model)	0.37	0.34	0.38	0.40	0.62	0.55	0.35	0.51

Note: *P < 0.05; **P < 0.01; ***P < 0.001. Non-standardised regression coefficients are presented

Statistically significant associations exist between nearly all independent variables and dependent variables included in Table 5. Thus, both activity type and social background variables (gender, age and educational level) are significantly associated with motives for PA, even when activity type has been controlled for social background characteristics and vice versa. The non-standardised regression coefficients with the highest magnitude can be found among the activity type variables. This could indicate that activity type is more strongly associated with motives for PA participation than social background when operationalised as gender, age and educational level.

When it comes to activity type differences, all results refer to running, which was chosen as the reference category. The results show that mastery and physical condition are more important motives in running than any other activity type. The same applies to psychological condition except for mental/flexibility/stability training (b = 0.13), to appearance except for fitness (b = 0.29), and to competition/ego with the exception of other ballgames (b = 1.39) and team ballgames (b = 0.89). By contrast, affiliation is generally more important in all other activity types compared to running. The same applies to others’ expectations, except for mental/flexibility/stability training (b = -0.04), and enjoyment, except for fitness (b = -0.09).

We find the largest differences in the magnitude of the regression coefficients according to activity type regarding physical condition, appearance and affiliation. With a coefficient below minus three compared to running, physical condition is a significantly less important motive for outdoor activities (b = -3.01), but also for activities on water (b = -2.44) and street sports (b = -2.38) with coefficients below minus two. Appearance is significantly less important for the same three activity types, but only with a coefficient below minus two compared to running for outdoor activities (b = -2.31). Regarding affiliation, two activity types have a coefficient higher than three compared to running, namely team ballgames (b = 3.55) and other ballgames (b = 3.45), while three have a coefficient above two, namely activities on water (b = 2.60), dance (b = 2.39) and outdoor activities (b = 2.16).

As concerns social background, women score higher than men for nearly all motives, with non-standardised beta coefficients between b = 0.13 regarding affiliation and b = 0.89 regarding psychological condition. The only exceptions are for competition/ego, which men rate as being significantly more important than women (b = -0.46), and for others’ expectations, for which no significant gender differences are observed.

The importance of physical condition (b_25-39 years_ = 0.40, b_40-59 years_ = 0.80, b_60+ years_ = 1.26), psychological condition (b_25-39 years_ = 0.57, b_40-59 years_ = 1.00, b_60+ years_ = 1.13) and enjoyment (b_25-39 years_ = 0.33, b_40-59 years_ = 0.62, b_60+ years_ = 0.65) increases with age, while the opposite holds for others’ expectations (b_25-39 years_ = -0.34, b_40-59 years_ = -1.05, b_60+ years_ = -1.20) and affiliation (b_25-39 years_ = -0.06, b_40-59 years_ = -0.28, b_60+ years_ = -0.32). Appearance is rated as significantly more important for people aged 25–39 years (b = 0.29), but less important for people aged 60 + years (b = -0.76), compared to people aged 15–24 years. Competition/ego is rated as significantly less important by all age categories compared to people aged 15–24 years, although this is most pronounced for people aged 40–59 years (b = -0.43) followed by people aged 60 + years (b = -0.35).

With increasing educational level, physical condition (b_secondary_ = 0.20, b_tertiary_ = 0.22) and enjoyment (b_secondary_ = 0.14, b_tertiary_ = 0.18) become more important motives, while mastery (b_secondary_ = -0.12, b_tertiary_ = -0.50), others’ expectations (b_secondary_ = -0.18, b_tertiary_ = -0.33) and affiliation (b_secondary_ = -0.07, b_tertiary_ = -0.12) become less important.

### Consistency of motives for PA participation across different activity types

Table 6 shows that the composition of individuals’ motives for PA participation are not consistent across activity types. When focusing on the 108,621 respondents who reported that they participate in two activity types or more at least once a week, the mean difference in their rating of each motive variates from 0.59 regarding mastery to 1.41 regarding others’ expectations.

**Table 6:** Mean differences and standard deviations in responses to eight motive items

	**Mastery**		**Physical condition**		**Affiliation**		**Psychological condition**		**Appearance**		**Others' expectations**		**Enjoyment**		**Competition/ego**		
	Mean diff	Std.dev	Mean diff	Std.dev	Mean diff	Std.dev	Mean diff	Std.dev	Mean diff	Std.dev	Mean diff	Std.dev	Mean diff	Std.dev	Mean diff	Std.dev	N
**Within individuals across activity types**																	
All individuals	0.59	(0.51)	1.10	(0.82)	1.19	(0.80)	1.14	(0.85)	1.09	(0.79)	1.41	(0.94)	1.12	(0.81)	1.25	(0.87)	108,621
**Across individuals within activity types**																	
Running	0.79	(0.67)	0.38	(0.42)	1.27	(0.64)	0.48	(0.45)	0.92	(0.75)	1.18	(0.60)	0.82	(0.67)	1.18	(0.73)	31,853
Walking and hiking	1.05	(0.77)	0.68	(0.53)	1.08	(0.71)	0.61	(0.47)	1.09	(0.74)	1.14	(0.58)	0.68	(0.49)	1.21	(0.61)	95,174
Biking	1.04	(0.77)	0.72	(0.54)	1.12	(0.79)	0.70	(0.52)	1.10	(0.76)	1.14	(0.58)	0.74	(0.53)	1.20	(0.71)	54,465
Fitness	0.83	(0.72)	0.34	(0.39)	1.18	(0.80)	0.44	(0.43)	0.95	(0.77)	1.18	(0.64)	0.81	(0.64)	1.16	(0.83)	58,703
Mental/flexibility/stability training	0.85	(0.67)	0.50	(0.49)	1.30	(0.67)	0.37	(0.44)	1.23	(0.77)	1.20	(0.58)	0.70	(0.53)	1.22	(0.59)	28,293
Team ballgames	0.87	(0.70)	0.77	(0.59)	0.58	(0.46)	0.76	(0.55)	1.09	(0.78)	1.10	(0.72)	0.56	(0.50)	0.95	(0.75)	9,501
Other ballgames	0.79	(0.64)	0.77	(0.58)	0.62	(0.45)	0.72	(0.54)	1.06	(0.82)	1.09	(0.77)	0.48	(0.44)	0.85	(0.73)	13,062
Gymnastics	0.94	(0.67)	0.58	(0.54)	1.06	(0.77)	0.59	(0.53)	1.13	(0.73)	1.15	(0.74)	0.75	(0.55)	1.16	(0.75)	13,785
Dance	0.96	(0.75)	0.88	(0.67)	0.96	(0.80)	0.71	(0.57)	1.18	(0.73)	1.18	(0.80)	0.56	(0.53)	1.24	(0.70)	9,235
Activities in water	1.06	(0.70)	0.73	(0.59)	1.17	(0.74)	0.59	(0.52)	1.15	(0.77)	1.19	(0.71)	0.61	(0.49)	1.22	(0.71)	18,814
Activities on water	0.85	(0.70)	0.92	(0.70)	0.84	(0.72)	0.69	(0.51)	1.14	(0.79)	1.14	(0.82)	0.47	(0.47)	1.18	(0.82)	4,453
Outdoor activities	0.99	(0.71)	1.00	(0.67)	0.94	(0.73)	0.74	(0.54)	1.13	(0.68)	1.09	(0.78)	0.60	(0.47)	1.17	(0.70)	14,938
Street sports	1.02	(0.73)	1.06	(0.69)	1.11	(0.75)	0.95	(0.70)	1.10	(0.82)	1.15	(0.75)	0.74	(0.53)	1.20	(0.76)	1,989

Our analysis reveals that for most motives, the mean differences between individuals within an activity type are higher than those within individuals across different activity types. This holds true for physical and psychological condition, others’ expectations, enjoyment and competition/ego. The only exception is mastery, where the mean differences are consistently higher across individuals within the same activity type. The results are inconclusive for affiliation and appearance.

## Discussion

Below we discuss our findings in the context of the existing literature and elaborate on the implications of these findings for research on motives and for activities, initiatives and programmes that seek to increase PA participation. Finally, we reflect on the limitations of our study and propose a research agenda on motives for PA participation.

### Prevalence of different motives for PA participation

Our study finds that psychological condition, physical condition and enjoyment are the most prevalent motives for PA participation. The least prevalent motives were identified as others’ expectations and competition/ego. These findings align well with findings in previous studies that rate physical condition, enjoyment and mastery as the most important motives for PA participation and others’ expectations as the least important motive [19, 24, 25]. The same can be said for psychological condition, which is also among the most prevalent motives in some studies [24, 25]. In our study, mastery is not rated as being as important as it is in other studies, which may be ascribable to the fact that a very large proportion of our responses to the motive items stem from people having replied with respect to activity types where mastery is generally not a very important motive. This is particularly true for walking and hiking (115,870 responses) and biking (56,891 responses), which jointly make up a little less than half of the total number of responses (Table 1).

### Associations between motives for PA participation, activity type and social background

Even though some motives for PA participation are generally more prevalent than others, our study underlines the relevance of examining motives according to activity type. This is evident when looking at a motive such as competition/ego, which is generally not an important motive for PA participation, but it is of central importance for team ballgames and other ballgames. The same can be said for affiliation. Affiliation is ranked fifth out of eight among the motive items in general prevalence; however, our analysis shows that while affiliation is a significantly less prevalent motive for activities such as running, biking and fitness, it is central to participation in team ballgames, other ballgames, activities on water and dance. These findings align well with previous studies having identified similar differences between team ballgames (e.g. football, hockey and Australian football) and exercise activities (e.g. fitness and running) [24–26]. Also, Chowdhury [26] identified tendencies that psychological condition is a prevalent motive among yoga participants, while affiliation, competition/ego and physical condition are less prevalent motives. We identify the same tendencies for participants in the activity type mental/flexibility/stability training, which includes yoga.

Our findings differ from previous research findings regarding mastery, which we find to be as prevalent a motive for exercise activities such as running and fitness as it is for team ballgames and other ballgames. In our study – like in other studies – exercise activities are thus found to be strongly motivated by psychological condition and physical condition, but our study also indicates that the role of skill improvement for exercise activities should not be neglected (e.g. running faster, lifting heavier weights).

Also, to our knowledge our study brings forth novel results regarding motives for activity types such as outdoor activities, activities on water, activities in water and street sports. As such, our study provides a more comprehensive picture of motives for PA participation across the complete range of leisure-time PA types.

The vast differences in motives according to activity types illustrate the need to study motives for PA participation depending on the activity types practised, because otherwise a potential recommendation from the general results would have been that, for instance, competition/ego and affiliation are of little importance for PA participation. As indicated by our results, the picture is much more complex.

In our study, we find that the non-standardised regression coefficients with the highest magnitude are found among the activity type variables rather than the social background variables, which indicates that activity type is more strongly associated with motives for PA participation than social background when operationalised as gender, age and educational level. This is an important finding, which should, however, not be taken to mean that social background is a negligible factor when studying motives for PA. Regarding gender, we find competition/ego to be a more prevalent motive for men compared to women and, conversely, we find women to be more motivated by appearance than men. These findings are consistent with previous studies [19, 24, 25]. In contrast to other studies [19, 24], we find that affiliation is a more prevalent motive for women compared to men. However, it should be noted that the non-standardised regression coefficient is rather small (b = 0.13), indicating only modest gender differences in this regard.

We also identified significant differences in motives according to age. The prevalence of physical condition and psychological condition increases with age, while the prevalence of affiliation and competition/ego decreases with age. These findings also mainly align with previous studies [24, 25]. However, our finding that the prevalence of others’ expectations decreases with age runs counter to the finding that the expectations of others are a more important motive for PA participation for middle-aged adults compared to young adults [24].

Looking at education, the differences are generally minor; however, our analysis does reveal that competition/ego and mastery are more prevalent motives for people with lower education, while physical condition and enjoyment are less prevalent. We were not able to identify studies that had examined the association between educational level and motives for PA participation, which indicates that these findings are novel.

### Consistency of individuals’ motives for PA participation

Our finding that motives for PA participation vary significantly according to activity type after controlling for social background variables (gender, age and education) can be interpreted as an indication that motives are not consistent within individuals across activity types. This is confirmed by our analysis of mean differences, which shows how the respondents’ ratings of motives vary between the different activity types. In fact, for most motive items, the ratings are less consistent within individuals across activity types than across individuals within the thirteen activity types included. To our knowledge, no other studies include similar consistency calculations, which underlines the novelty of this finding. It is important because it shows that people’s rationales for PA participation will vary depending on the activity type practised, e.g. whether they play team ballgames, practise outdoor activities or go for a run.

### Implications for research on motives for PA participation

Our findings have implications for research on motives for PA participation. One implication is that motives should be studied according to the activity type practised. The lack of consistency in individuals’ motives for PA participation and the substantial differences in motives between the thirteen activity types included in our study illustrate this.

Our findings can also provide a basis for discussing whether motives should be viewed as consistent constructs tied to the individual, or if they are, in fact, dynamic constructs, depending on, among other things, the activity type practised by an individual. We identified a lack of consistency in individuals’ motives for PA participation and found that motives vary both within individuals across activity types and across individuals within activity types. Though our data are cross-sectional, this may indicate that motives develop in the relation between an individual and the activity type practised. This seems to challenge the most common way of thinking about motives as an independent variable that affect the dependent variable, in this case the choice of an activity type. Based on our findings, one could hypothesise that the relationship between motives and PA participation is more likely to resemble a bidirectional relationship rather than a unidirectional one.

### Implications for activities, initiatives and programmes that seek to increase PA participation

Studies on motives for PA participation often suggest that sport and health professionals should use their knowledge about differences in motives according to activity type and social background to match an individual or a specific group of individuals with selected types of PA [10, 13–15]. For example, young men could be directed towards team ballgames, because competition/ego is positively associated with participation in this activity type and with the gender and age characteristics of this group.

The suggested matching approach might still have relevance, but it is associated with the risk of neglecting the bidirectional relationship that we – based on our findings – argue is likely to exist between an individual’s motive composition and choice of activity type. Accordingly, we argue that activities, initiatives and programmes aimed at increasing PA participation should not only or not mainly rely on matching individuals and activity types based on knowledge about the motive composition of an individual or a social group, since motives are likely to develop in the interplay between the individual and the activity type practised.

Our study challenges the idea that individuals have a universal set of motives for PA participation. Individuals who are inactive and have limited experience with PA are unlikely to be able to validly rate different motives to start and maintain regular PA participation. As a result, it seems more effective to design PA programmes, activities and initiatives based on an understanding of how to tailor different types of PA to people with lower capabilities to engage in PA and to take into consideration both the opportunities and often multifaceted barriers faced by people who are less physically active. This is in line with the broad focus included in the behaviour change wheel suggested by Michie and colleagues [30] as an instrument to develop programmes, activities and initiatives to increase PA participation in general as well as specifically among people with sedentary lifestyles.

### Limitations and research agenda

In our operationalisation of motives for PA, we were inspired by the PALMS framework. However, because of our decision to examine motives in relation to a wide range of activity types, we only included one motive item per dimension in the PALMS framework. As a result, we might not have examined the full complexity of each dimension. However, the consistency of our findings regarding both the relative importance of motives in general and according to activity type and social background serves as an indication that our operationalisations were valid.

When operationalising participation in the activity types included in our analyses, participants were coded as those who participated at least once a week. With this perspective, we neglect the potential association that could exist between motives on the one hand and frequency and duration of participation on the other. This represents an important future research agenda.

Because our study is cross-sectional, we examine associations rather than causal relationships. As a result, our consistency analysis might indicate the existence of a bidirectional rather than a unidirectional relationship between motives and activity types, but time series data are needed to examine this claim empirically.

Departing from these limitations, we propose a research agenda with a need for both quantitative and qualitative studies. First, quantitative studies of a longitudinal type are needed to examine changes in motives over time and within individuals across activities. This could be in the form of panel studies in which PA participation and motives are examined using the same questions among the same group of respondents with regular intervals. Next, qualitative studies (e.g. interview studies) are well-suited to shed light on the causal mechanisms that can help explain the associations identified between motives on the one hand and social background and activity type on the other. Finally, future studies should consider the role of frequency and duration of participation in the various activity types as this could provide novel knowledge on this topic.

## Conclusions

In this article we examined the prevalence of different motives for PA participation, the association between activity type and social background on the one hand and motives on the other as well as the consistency of individuals’ motives across different activity types. We identified large differences in the prevalence of different motives for PA participation, found significant associations between motives and activity type in particular, but also social background, and found that individuals’ motives are not consistent across different activity types. This indicates that motives are not consistent constructs. Rather, they are likely to develop in an interplay between the individual and the activity type practised.

### Supplementary Information

**Additional file 1: Supplementary Table 1. **Mean values and standard deviations for the eight motive items calculated within the independent variable categories (*N*=385,631)

## Data Availability

Due to data protection regulation and the sensitive nature of parts of the data collected in the survey study, the data is not publicly available. For any queries regarding the data, please contact Karsten Elmose-Østerlund at kosterlund@health.sdu.dk.
