# Supplementary material for: Motives for leisure-time physical activity participation: an analysis of their prevalence, consistency and associations with activity type and social background
Source: BMC Public Health. 2023 Dec 2;23:2399. doi: 10.1186/s12889-023-17304-0 (PMC10693154; doi:10.1186/s12889-023-17304-0)
Supplement: Supplementary file 1 — Additional file 1: Supplementary Table 1. Mean values and standard deviations for the eight motive items calculated within the independent variable categories (N=385,631) [file 12889_2023_17304_MOESM1_ESM.docx]

Supplementary Table 1. Mean values and standard deviations for the eight motive items calculated within the independent variable categories (N=385,631).

|  | **Mastery** | | **Physical condition** | | **Affiliation** | | **Psychological condition** | | **Appearance** | | **Others' expectations** | | **Enjoyment** | | **Competition/ego** | |
| --- | --- | --- | --- | --- | --- | --- | --- | --- | --- | --- | --- | --- | --- | --- | --- | --- |
|  | Mean | Std.dev. | Mean | Std.dev. | Mean | Std.dev. | Mean | Std.dev. | Mean | Std.dev. | Mean | Std.dev. | Mean | Std.dev. | Mean | Std.dev. |
| **Activity type** |  |  |  |  |  |  |  |  |  |  |  |  |  |  |  |  |
| Running | 4.11 | (1.04) | 4.77 | (0.56) | 2.57 | (1.43) | 4.68 | (0.66) | 3.91 | (1.19) | 2.51 | (1.32) | 4.13 | (1.06) | 3.32 | (1.39) |
| Walking and hiking | 3.19 | (1.31) | 4.41 | (0.90) | 3.33 | (1.31) | 4.50 | (0.82) | 3.23 | (1.33) | 2.46 | (1.28) | 4.40 | (0.89) | 2.38 | (1.35) |
| Biking | 3.24 | (1.30) | 4.39 | (0.90) | 2.89 | (1.37) | 4.41 | (0.88) | 3.23 | (1.34) | 2.43 | (1.28) | 4.34 | (0.92) | 2.66 | (1.39) |
| Fitness | 3.99 | (1.11) | 4.80 | (0.52) | 2.94 | (1.42) | 4.71 | (0.63) | 3.95 | (1.23) | 2.61 | (1.34) | 4.10 | (1.05) | 3.00 | (1.42) |
| Mental/flexibility/stability training | 4.11 | (1.09) | 4.67 | (0.70) | 2.63 | (1.46) | 4.77 | (0.58) | 3.23 | (1.45) | 2.38 | (1.33) | 4.43 | (0.89) | 2.27 | (1.36) |
| Team ballgames | 3.93 | (1.12) | 4.24 | (0.97) | 4.58 | (0.73) | 4.30 | (0.94) | 3.15 | (1.34) | 3.45 | (1.31) | 4.62 | (0.75) | 3.91 | (1.21) |
| Other ballgames | 4.09 | (1.02) | 4.27 | (0.96) | 4.50 | (0.77) | 4.41 | (0.90) | 2.88 | (1.33) | 3.12 | (1.34) | 4.69 | (0.65) | 4.06 | (1.12) |
| Gymnastics | 3.82 | (1.16) | 4.61 | (0.80) | 3.79 | (1.31) | 4.59 | (0.80) | 3.45 | (1.35) | 2.78 | (1.37) | 4.37 | (0.94) | 2.73 | (1.38) |
| Dance | 3.95 | (1.22) | 4.19 | (1.11) | 4.01 | (1.25) | 4.47 | (0.91) | 3.39 | (1.39) | 2.92 | (1.43) | 4.63 | (0.76) | 2.62 | (1.42) |
| Activities in water | 3.52 | (1.28) | 4.44 | (0.94) | 3.48 | (1.38) | 4.58 | (0.80) | 3.27 | (1.38) | 2.71 | (1.38) | 4.55 | (0.78) | 2.65 | (1.41) |
| Activities on water | 4.03 | (1.10) | 3.94 | (1.15) | 4.10 | (1.11) | 4.45 | (0.86) | 2.80 | (1.38) | 2.98 | (1.40) | 4.70 | (0.66) | 3.08 | (1.44) |
| Outdoor activities | 3.40 | (1.22) | 3.69 | (1.20) | 3.85 | (1.19) | 4.32 | (0.92) | 2.57 | (1.32) | 2.86 | (1.34) | 4.57 | (0.76) | 2.63 | (1.37) |
| Street sports | 3.89 | (1.26) | 3.69 | (1.26) | 3.76 | (1.35) | 3.89 | (1.19) | 3.08 | (1.38) | 3.27 | (1.38) | 4.38 | (0.92) | 3.26 | (1.43) |
| **Social background** |  |  |  |  |  |  |  |  |  |  |  |  |  |  |  |  |
| Gender: man | 3.57 | (1.26) | 4.36 | (0.95) | 3.24 | (1.40) | 4.39 | (0.89) | 3.15 | (1.37) | 2.62 | (1.33) | 4.27 | (0.96) | 2.96 | (1.43) |
| Gender: woman | 3.64 | (1.28) | 4.56 | (0.82) | 3.23 | (1.44) | 4.65 | (0.70) | 3.53 | (1.34) | 2.57 | (1.35) | 4.43 | (0.89) | 2.59 | (1.42) |
| Age: 15-24 | 3.80 | (1.27) | 4.20 | (1.05) | 3.42 | (1.41) | 4.26 | (0.98) | 3.51 | (1.35) | 3.13 | (1.32) | 4.13 | (1.03) | 3.13 | (1.46) |
| Age: 25-39 | 3.64 | (1.34) | 4.40 | (0.94) | 3.26 | (1.46) | 4.50 | (0.82) | 3.63 | (1.34) | 2.83 | (1.36) | 4.30 | (0.94) | 2.85 | (1.50) |
| Age: 40-59 | 3.49 | (1.30) | 4.50 | (0.86) | 3.14 | (1.45) | 4.60 | (0.74) | 3.46 | (1.35) | 2.46 | (1.32) | 4.41 | (0.89) | 2.63 | (1.43) |
| Age: 60+ | 3.63 | (1.20) | 4.58 | (0.77) | 3.24 | (1.38) | 4.60 | (0.75) | 3.11 | (1.37) | 2.42 | (1.29) | 4.42 | (0.90) | 2.69 | (1.38) |
| Education: primary | 3.74 | (1.22) | 4.33 | (0.98) | 3.37 | (1.38) | 4.40 | (0.92) | 3.36 | (1.36) | 2.83 | (1.36) | 4.26 | (0.99) | 2.99 | (1.43) |
| Education: secondary | 3.66 | (1.24) | 4.48 | (0.87) | 3.25 | (1.40) | 4.55 | (0.78) | 3.38 | (1.35) | 2.59 | (1.32) | 4.37 | (0.91) | 2.82 | (1.42) |
| Education: tertiary | 3.49 | (1.32) | 4.53 | (0.84) | 3.16 | (1.46) | 4.59 | (0.75) | 3.36 | (1.39) | 2.48 | (1.33) | 4.39 | (0.91) | 2.58 | (1.43) |
